# The protective role of human ghrelin in sepsis: Restoration of CD4 T cell proliferation

**DOI:** 10.1371/journal.pone.0201139

**Published:** 2018-07-27

**Authors:** Mian Zhou, Monowar Aziz, Manhendar Ochani, Weng-Lang Yang, Archna Sharma, Ping Wang

**Affiliations:** 1 Center for Immunology and Inflammation, The Feinstein Institute for Medical Research, Manhasset, New York, United State of America; 2 Department of Surgery and Molecular Medicine, Donald and Barbara Zucker School of Medicine at Hofstra/Northwell, Manhasset, New York, United State of America; University of Kentucky, UNITED STATES

## Abstract

Decrease of CD4 T cell numbers causes immunosuppression in sepsis. We previously showed the beneficial role of ghrelin in sepsis. We hypothesize that the protective outcome of ghrelin in sepsis is mediated partially through the restoration of CD4 T cells’ proliferation. Sepsis was induced in mice by cecal ligation and puncture (CLP). The percentage of CD4 T cells in spleen was assessed by flow cytometry and their proliferation was determined by carboxyfluorescein succinimidyl ester (CSFE). Compared to sham mice, the percentages of splenic CD4 T cells were reduced by 20%, 21%, and 29% at day 1, 2 and 3 after CLP, respectively. Human ghrelin was given to 3 day septic mice by *s*.*c*. injection at 5 and 24 h after CLP. Treatment with ghrelin restored the loss of CD4 T cells by increasing their proliferation in septic mice. The expression of cyclin D1 and B1 was significantly increased, while the expression of p57 was decreased in ghrelin-treated mice compared to vehicle-treated mice in sepsis. Treatment with human ghrelin significantly increased the p-AKT levels in the spleen compared to vehicle-treated septic mice. Human ghrelin plays an important role in reestablishing the proliferation of CD4 T cells and serves as a promising therapeutic agent in sepsis.

## Introduction

Sepsis remains a major public health concern world-wide. Sepsis occurs due to a dysregulated host immune response to an invading pathogen [1, 2]. Interaction of pathogen-associated molecular patterns (PAMPs) with Toll-like receptors (TLRs) leads to cytokine storm causing exaggerated inflammation [1, 3]. As the pro-inflammatory cytokines are responsible for triggering the inflammation in sepsis, the therapeutic benefits of anti-cytokine regimens have been demonstrated in animal models, nonetheless no remarkable achievements are yet to be reported [4]. Although the early onset of hypercytokinemia can be opposed by anti-inflammatory cytokines, prolonged persistence of anti-inflammatory cytokines may lead to an immunosuppressive condition [2]. Patients who survive severe sepsis often display suppressed immune function, which is characterized by an inability to respond to antigens, and thus become susceptible to secondary infections [5]. In fact, the compromised host immunity during protracted sepsis is associated with increased mortality rate in sepsis [6, 7]. An important mechanism of immunosuppression is the extensive depletion of immune cell population and alterations in the lymphocyte functions [8, 9]. A finely tuned balance between pro- and anti-inflammatory events is important for better prognosis in sepsis. As most deaths occur during the prolonged hypo-immune phase of sepsis, reversal of this immune deficiency generates a better survival outcome. Therefore, development of an effective therapeutic potential for maintaining normal immune function during sepsis is crucial.

CD4 T cells are a subgroup of lymphocytes that play a central role in immune protection. CD4 T cells help B cell maturation, activate cytotoxic T cells and macrophages, and recruit neutrophils to the sites of infection by releasing T cell cytokines [8–10]. Sepsis causes a significant reduction in the numbers of CD4 T cells, which affects host response to infection [8, 11, 12]. In animal model of sepsis and postmortem tissue samples from septic patients, apoptosis has a major impact on the depletion of immune cells [8, 9, 13, 14]. Besides lymphocyte apoptosis, decreased rate of CD4 T cell proliferation has been demonstrated in sepsis [8, 12, 15]. Therefore, both apoptosis and impaired proliferation of CD4 T cells are the key events to cause their reduction in numbers in sepsis.

Human ghrelin is a 28-amino acid secretory peptide, predominantly produced by the stomach [16]. Ghrelin binds to growth hormone secretagogue receptor (GHSR)-1a to promote the release of growth hormone (GH) [16]. Ghrelin promotes endocrine, as well as non-endocrine activities, including anti-inflammation and organ protection [17, 18]. Ghrelin levels are decreased in the circulation during early as well as late phase of sepsis [19]. Correspondingly, ghrelin expression in the stomach and lungs are also significantly reduced in sepsis [18, 19]. Interestingly, in aged animals, the decrease of ghrelin expression is much higher than young septic animals [19, 20]. Moreover, the expression of ghrelin receptor GHSR-1 is not decreased in young animals, while it is downregulated in aged animals following sepsis [20]. The lower levels of ghrelin and ghrelin receptor expression in aged septic animals correlate with severe organ damage and higher mortality rate as compared to young animals [20, 21].

GH increases the sensitivity of ghrelin receptor in aged animals [20]. We previously showed ghrelin in combination with GH significantly restored immune response in aged septic rats [22]. The increased immune response following treatment with ghrelin and GH in aged septic animals were correlated with increased expression of cytokines to *ex vivo* LPS stimulation, reduced apoptosis of CD4 T cells, reduced frequencies of regulatory T cells (T_reg_), and decreased expression of inhibitory co-receptors such as programmed death 1 (PD1) receptor, programmed death-ligand 1 (PD-L1) and cytotoxic T lymphocyte antigen 4 (CTLA4) [22]. Since the expression of ghrelin receptor is not decreased in young animals after sepsis [20, 22], the ghrelin-mediated functions through its receptor could be normal and thus precludes the need of additional growth hormone for the treatment of young septic mice.

Ghrelin has been shown to promote thymopoiesis and T cell proliferation in the thymus [23, 24]. Ghrelin receptor is expressed in a variety of immune cells, including T cells, monocytes and dendritic cells [23–25]. Thus, besides CD4 T cells ghrelin may also regulate macrophage and dendritic cell function. Several studies demonstrated that the T cells can produce ghrelin as an autocrine or paracrine ligand to regulate immune microenvironment [23–25].

In the current study, we aim to investigate the role of ghrelin for promoting the proliferation of CD4 T cells after sepsis. Our data clearly demonstrated the restoration of CD4 T cell proliferation in young septic mice following treatment with ghrelin by upregulating the expression of cell cycle positive regulators and downregulating the expression of a cell cycle negative regulator. Thus, ghrelin benefits sepsis partially through the restoration of CD4 T cell proliferation.

## Materials and methods

### Animal model of sepsis

Male 10-week-old C57BL/6 mice were obtained from Charles River Laboratories (Wilmington, MA). All animals were housed in a temperature-controlled room under a 12 h light-dark cycle, fed a standard laboratory mouse diet and provided drinking water *ad libitum*. Mice were allowed to acclimate to the environment for at least 7 days before being used for experiments. Sepsis was induced in mice by cecal ligation and puncture (CLP) [26]. The mice were anesthetized by 2% isoflurane inhalation. The abdomen was shaved and washed with 10% povidone iodine. A 2-cm midline laparotomy was performed to expose the cecum and ligate 1 cm from its tip with a 3–0 silk suture. The cecum was punctured once using a 22-gauge needle and then returned to the peritoneal cavity. The laparotomy site was closed in layer with 4–0 silk suture. The CLP animals were immediately resuscitated with 1 ml normal saline containing Primaxin (Merck, Whitehouse Station, NJ) as antibiotic at a dose 0.5 mg/kg body weight subcutaneously (*s*.*c*.). Sham operated animals underwent incision of the abdomen, but their cecum was neither ligated nor punctured. Spleen was harvested at day 1, 2 and 3 after CLP. All experiments were performed following the guidelines for the use of experimental animals as demonstrated by the National Institutes of Health, and the study was approved by the Institutional Animal Care and Use Committee (IACUC) of the Feinstein Institute for Medical Research. Humane care and proper use of anesthetics were provided. The animals were monitored twice a day for signs of sepsis severity and animal welfare. After CLP operation, we used warming blanket and subcutaneous fluid resuscitation to prevent hypothermia and dehydration. Humane endpoints were considered for all experiments involving animals. At the experimental endpoint, CO_2_ inhalation the most widely used method for euthanizing animals were used. The main criteria for euthanasia were: minimal or absence of movement, hunched or recumbent posture, minimal or no response to stimulus and body condition scoring (BCS) of 2 or less. No mice was euthanized before meeting criteria for euthanasia. The IACUC approval included the sacrifice of the mice.

### Administration of human ghrelin

Human ghrelin (Phoenix Pharmaceuticals, Belmont, CA) was dissolved in normal saline. Human ghrelin at a dose 2 nmol/mouse/injection was administrated *s*.*c*. at 5 and 24 h after CLP. The vehicle treated animals received same volume of normal saline *s*.*c*.. After 3 days of CLP, spleen was harvested for various analyses.

### Isolation of splenocytes

Animals were euthanized at 1, 2 and 3 days after CLP for collecting the spleen. Splenic single cell suspensions were obtained by gentle grinding between frosted glass slides in RPMI 1640 medium (Life Technologies, Grand Island, NY), followed by passage through a 70 μm nylon mesh (Fisher Scientific, Suwanee, GA). Red blood cells (RBC) were lysed using RBC lysing buffer (BD Biosciences, San Jose, CA). After centrifugation at 280 g for 5 min, the cell pellets were re-suspended in RPMI 1640 medium containing 10% heat-inactivated fetal bovine serum, 2 mM L-glutamine, 100 U/ml penicillin, 100 μg/ml streptomycin, 10 mM HEPES and 0.5 μM 2-mercaptaethanol, which we named complete RPMI 1640 medium for cell culture and proliferation assay.

### Flow cytometric detection of CD4 T cells in spleen

Splenocytes were isolated from mice at day 1, 2 and 3 of CLP. A total of 1 × 10^6^ single cell suspensions of splenocytes from each mouse were stained with APC-anti-mouse CD4 Ab (Clone RM4-5; Biolegend, San Diego, CA). A total of 30,000 events were acquired using a BD LSR Fortessa Flow Cytometry Analyzer (BD Biosciences) and data were analyzed by FlowJo software (Tree Star, Ashland, OR).

### Cell proliferation assay

Splenocytes were isolated from mice at day 1, 2 and 3 after CLP. A total of 1 × 10^6^ single cell suspensions of splenocytes were stained with 0.25 μM CellTrace carboxyfluorescein succinimidyl ester (CFSE) cell proliferation reagent (Thermo Fisher Scientific, Bridgewater, NJ) in 37°C for 8 min and then the reaction was stopped by adding 5 × volumes of RPMI 1640 medium. The cells were centrifuged at 280 g for 5 min and suspended in complete RPMI 1640 medium before plating them into anti-CD3/CD28 Ab (1.5 μg/ml each Ab, BioLegend)-coated 24-well plates. After 80 h of incubation, the cells were stained with APC anti-mouse CD4 Abs (Clone RM4-5; BioLegend). The labeled cells were acquired for flow cytometric analysis using a BD LSR Fortessa flow cytometry analyzer (BD Biosciences) and data were analyzed by FlowJo software (Tree Star). Freshly isolated splenocytes following labeled with APC-anti-mouse CD4 Abs (Clone RM4-5; BioLegend) and CFSE served as a non-dividing control. Fc blocker (anti-mouse CD16/32, clone 93, BioLegend) were used to exclude non-specific binding of Abs on cell surface.

### Western blot analysis

Spleen tissues were homogenized and lysed in RIPA buffer containing 10 mM Tris buffered saline pH 7.5, 1% Triton x-100, 1 mM EDTA, 1 mM EGTA, 2 mM Na orthovanadate and cocktail of protease inhibitors (Roche Diagonstics, Indianapolis, IN). Protein concentration was determined by the protein assay reagent (Bio Rad, Hercules, CA). Protein lysates in loading buffer were electrophoresed on 4–12% Bis-Tris NuPAGE gel (Life Technologies, Grand Island, NY) and then transferred to nitrocellulose membranes. The blots were reacted with anti-mouse Fas (Proteintech, Chicago, IL), Fas-L, cleaved caspase-8 (Cell Signaling Technology, Danvers, MA), cyclin D1, cyclin B1 (Proteintech), p57 (Abcam, Cambridge, MA) and phosphorylated AKT (p-AKT, Cell Signaling Technology) primary Abs overnight at 4°C. Infrared dye-labeled secondary Abs (Li-Cor, Lincoln, NE) were then applied and the target bands were revealed and analyzed by using an Odyssey infrared imaging system (Li-Cor, Lincoln, NE). All immunoblots were stripped-out and re-probed with anti-β-actin Abs (Sigma, St.Louis, MO), which served as loading/internal control.

### Statistical analysis

All data were expressed as mean ± SEM and compared by one-way ANOVA and student-Newman-Keuls (SNK) method. *Student t-test* was performed when only two groups were compared. Differences in values were considered significant when the *P* value represented <0.05.

## Results

### Sepsis causes a decrease in CD4 T cells in the spleen

**Fig 1:**
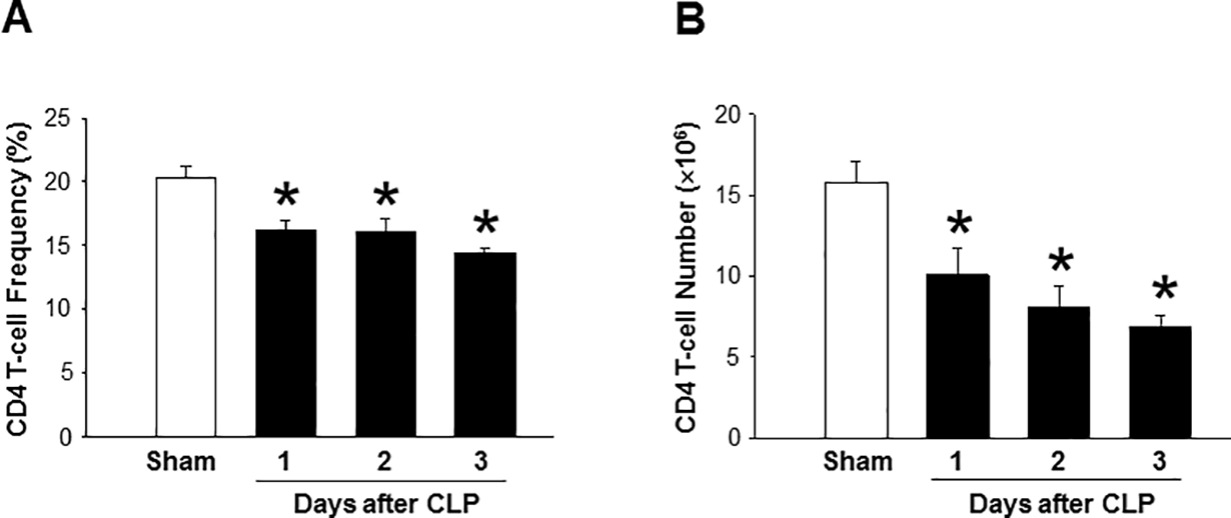
Sepsis decreases the frequencies of CD4 T cells. Mice were subjected to CLP or sham operation and spleens were harvested at day 1, 2 and 3 after CLP. Isolated splenocytes were stained for APC- anti-mouse CD4 Abs. (A) The percentages of CD4 T cells were assessed by flow cytometry and (B) total CD4 T cells in the spleen were calculated by multiplying total splenic cells. Data are expressed as mean ± SEM (n = 4–5 mice/group). *P < 0.05 vs. sham mice. CLP, cecal ligation and puncture; CD, cluster of differentiation.

Spleen was harvested from mice at day 1, 2 and 3 after CLP. The percentages of CD4 T cells in spleen were significantly decreased by 20%, 21%, and 29% at day 1, 2 and 3 after CLP, respectively, as compared to sham-operated mice (Fig 1A). Similarly, total numbers of CD4 T cells in the spleen were also markedly reduced by 36%, 49% and 56% at day 1, 2 and 3 after CLP, respectively, as compared to sham animals (Fig 1B).

### Impairment of ex vivo proliferation of splenic CD4 T cells of septic mice

**Fig 2:**
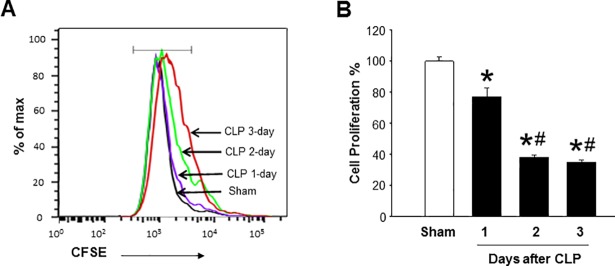
Sepsis impairs the proliferation of CD4 T cells *ex vivo*. Mice were subjected to CLP or sham operation and spleens were harvested at day 1, 2 and 3 after CLP. Isolated splenocytes were labeled with CFSE (5 μM) and stained with APC- anti-mouse CD4 Abs after the culture. The frequencies of proliferated cells were calculated and the sham group was normalized as 100%. Data are expressed as mean ± SEM (n = 4–5 mice/group). *P < 0.05 vs. sham and ^#^P < 0.05 vs. day 1 after CLP. CLP, cecal ligation and puncture; CFSE, carboxyfluorescein succinimidyl ester; CD, cluster of differentiation.

The CD4 T cell pool is maintained through their proliferation [27]. Here, we performed an *ex vivo* proliferation assay of the CD4 T cells isolated from sham and various time points of CLP mice. CFSE is a membrane permeable fluorescent dye. When a CFSE-labeled cell divides, the intensity of CFSE fluorescence in daughter cells reduces to half. Thus, each cell division can be assessed by measuring the corresponding decrease in CFSE fluorescence. CFSE-labeled splenocytes were collected from the culture and stained with anti-CD4 Abs. The cell proliferation percentages were calculated as the percentages of the decrease of CFSE fluorescence from sham mice. The CFSE fluorescence in sham group was normalized as 100%. The splenic CD4 T cell proliferation rate (as expressed by percentage) was markedly decreased by 23% at day 1 and further reduced by 61% and 65% at day 2 and 3 after CLP, respectively, as compared to sham mice (Fig 2A and 2B).

### Altered expression of cell cycle regulatory proteins in spleen during sepsis

**Fig 3:**
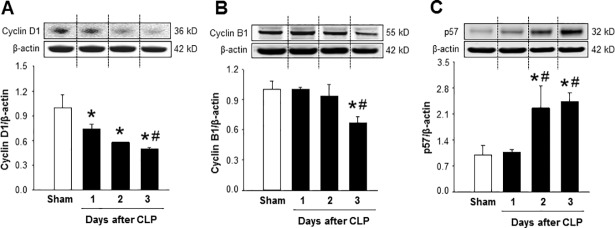
Sepsis alters the expression of cell cycle proteins in the spleen. Mice were subjected to CLP or sham operation and spleens were harvested at day 1, 2 and 3 after CLP. Total protein was extracted from spleen and the expression of (A) cyclin D1, (B) cyclin B1, and **(C)** p57 were measured by western blotting. Data are expressed as mean ± SEM (n = 4–5 mice/group). *P < 0.05 vs. sham and ^#^P < 0.05 vs. day 1 after CLP. The dotted lines on the representative Western blot reflect the corresponding groups as shown in the bar diagram bellow. CLP, cecal ligation and puncture.

Cell proliferation is regulated by cell cycle regulatory proteins [28]. Cylin D1 determines whether or not a cell enters into the cell cycle from resting state, G_0_ phase [28]. We noticed significant decrease in the expression of cyclin D1 in spleen tissues by 26% at day 1 after CLP, while its expression was further decreased by 42% and 50% at day 2 and 3 after sepsis as compared to sham-operated mice, respectively (Fig 3A). Cyclin B1 determines whether or not a cell enters into the mitosis and to be divided [28]. We found that the expression of Cyclin B1 in spleen was not altered at day 1 and 2 after CLP, while its expression was markedly reduced by 33% at day 3 after CLP compared to sham-operated mice (Fig 3B). On the other hand, the expression of cell cycle negative regulator p57 in spleen was significantly elevated by 126% and 143% at day 2 and 3 after sepsis, respectively, as compared to sham mice (Fig 3C). These results clearly indicated that impairment of CD4 T cell proliferation in late sepsis was associated with the decrease of cell cycle positive regulators cyclin D1 and B1 and increase of cell cycle negative regulatory protein p57.

### Sepsis triggers the expression of apoptosis inducing proteins in the spleen

**Fig 4:**
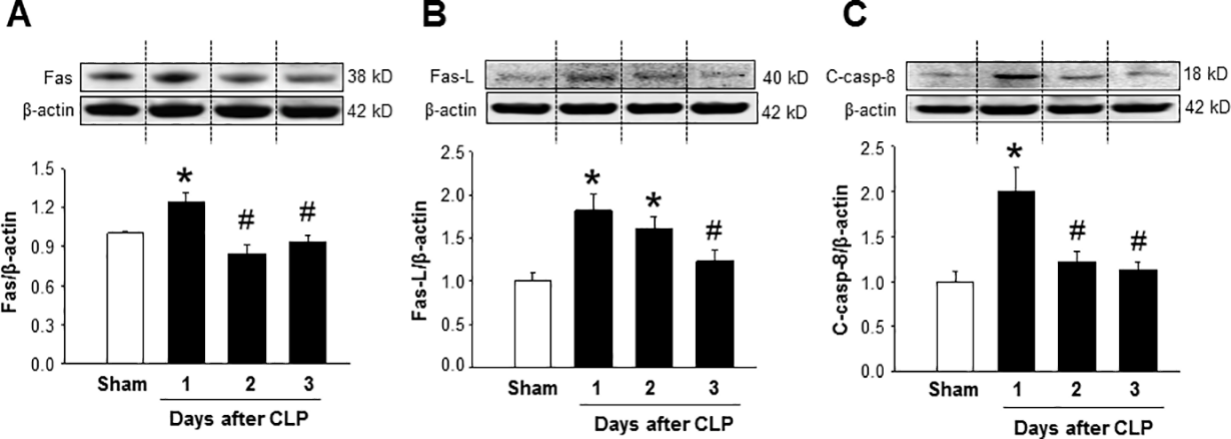
Sepsis induces the expression of apoptosis-related proteins in the spleen. Mice were subjected to CLP or sham operation and spleens were harvested at day 1, 2 and 3 after CLP. Total proteins were extracted from spleen and the expression of (A) Fas, (B) Fas-L and (C) cleaved caspase-8 were measured by western blotting. Data are expressed as mean ± SEM (n = 4–5 mice/group). *P < 0.05 vs. sham and ^#^P < 0.05 vs. day 1 after CLP. The dotted lines on the representative Western blot reflect the corresponding groups as shown in the bar diagram bellow. CLP, cecal ligation and puncture.

One of the major mechanisms of decreasing the frequencies of CD4 T cells during sepsis is through their apoptosis [12, 13, 22]. Fas and Fas-L pathway is directly linked with lymphocyte apoptosis [10, 15]. To assess the expression of Fas and Fas-L proteins at different time points after CLP, spleen tissues were harvested from mice at 1, 2 and 3 days after CLP. We noticed a 25% increase of the expression of Fas at its protein level in the spleen of septic mice at day 1 after CLP compared to sham mice, while its expression returned to nearly sham level at day 2 and 3 after CLP (Fig 4A). Similarly, at day 1 of CLP the expression of Fas-L was increased to its highest level by 82% in the spleen as compared to that of sham-operated mice (Fig 4B). The expression of Fas-L was found to increase by 61% and 24% at day 2 and 3 after CLP, respectively, compared to sham mice (Fig 4B). Upregulation of Fas and Fas-L expression cleaves downstream caspase-8 which leads to increase cellular apoptosis [29]. Our data showed a definitive correlation between upregulation of Fas and Fas-L expression and activation of caspase-8 in the spleen in sepsis. As shown in Fig 4C, cleaved caspase-8 levels in the spleen was significantly increased by 100% at day 1 after CLP compared to sham mice. Similar to Fas and Fas-L findings, the levels of cleaved caspase-8 in the spleen at day 2 and 3 after CLP was also not noticeably up-regulated compared to sham mice (Fig 4C). Thus, the increased expression of apoptosis inducing proteins at the early time point (day 1) of sepsis might lead to CD4 T cell apoptosis in the spleen as reported in our previous study [22]. Since the expression of apoptosis inducing proteins did not alter at day 2 and 3 of sepsis, the prolonged decreased levels of CD4 T cells in the spleen could be due to their proliferation defect.

### Ghrelin prevents the loss of CD4 T cells in spleen during sepsis

**Fig 5:**
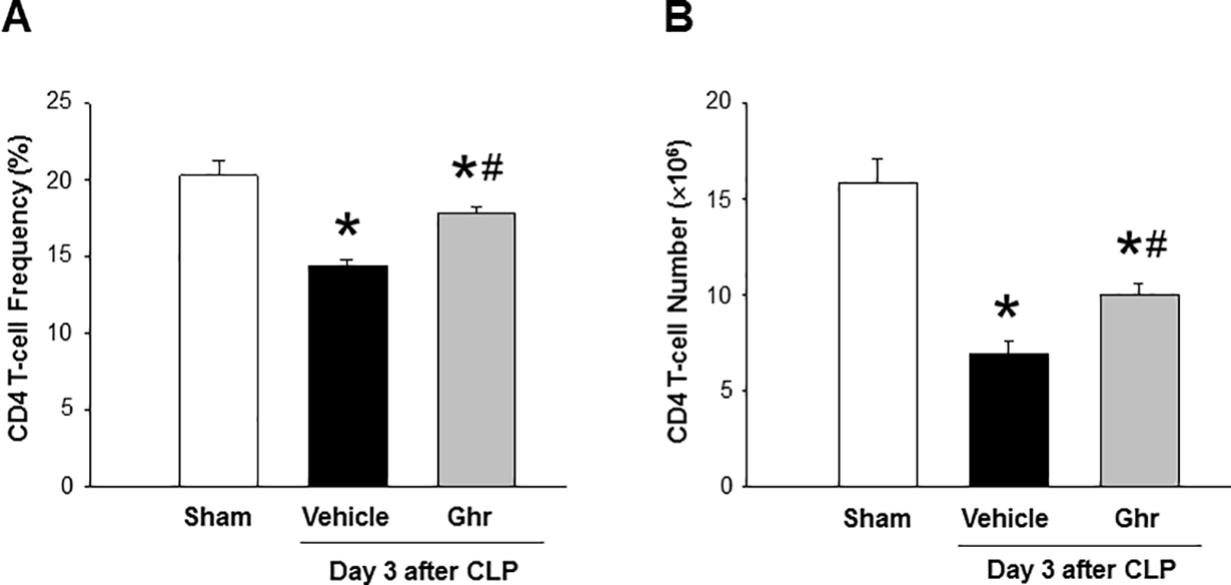
Ghrelin prevents the loss of CD4 T cells after sepsis. Mice were subjected to sham operation or CLP with vehicle (normal saline) or ghrelin (2 nm/mouse) injections at 5 and 20 h after CLP. Spleen was harvested at day 3 after CLP. Isolated splenocytes were stained for APC- anti-mouse CD4 Abs. (A) The percentages of CD4 T cells in the splenocytes were assessed by flow cytometry and (B) total CD4 T cells were calculated by multiplying total splenic cells. Data are expressed as mean ± SEM (n = 4–5 mice/group). *P < 0.05 vs. sham mice. CLP, cecal ligation and puncture.

Since the proliferation of splenic CD4 T cells of day 3 of CLP-operated mice was impaired most noticeably, we treated mice with ghrelin and investigated the effect of ghrelin on the *ex vivo* proliferation of CD4 T cells isolated from spleen of day 3 of CLP mice. We found that the CD4 T cell frequencies and their total numbers in the spleen were significantly reduced at day 3 after CLP compared to sham mice (Fig 5A and 5B). However, treatment of septic mice with ghrelin significantly restored the CD4 T cell frequencies and total CD4 T cell numbers in the spleen of day 3 CLP-operated mice (Fig 5A and 5B).

### Ghrelin restores splenic CD4 T cell proliferation in sepsis

**Fig 6:**
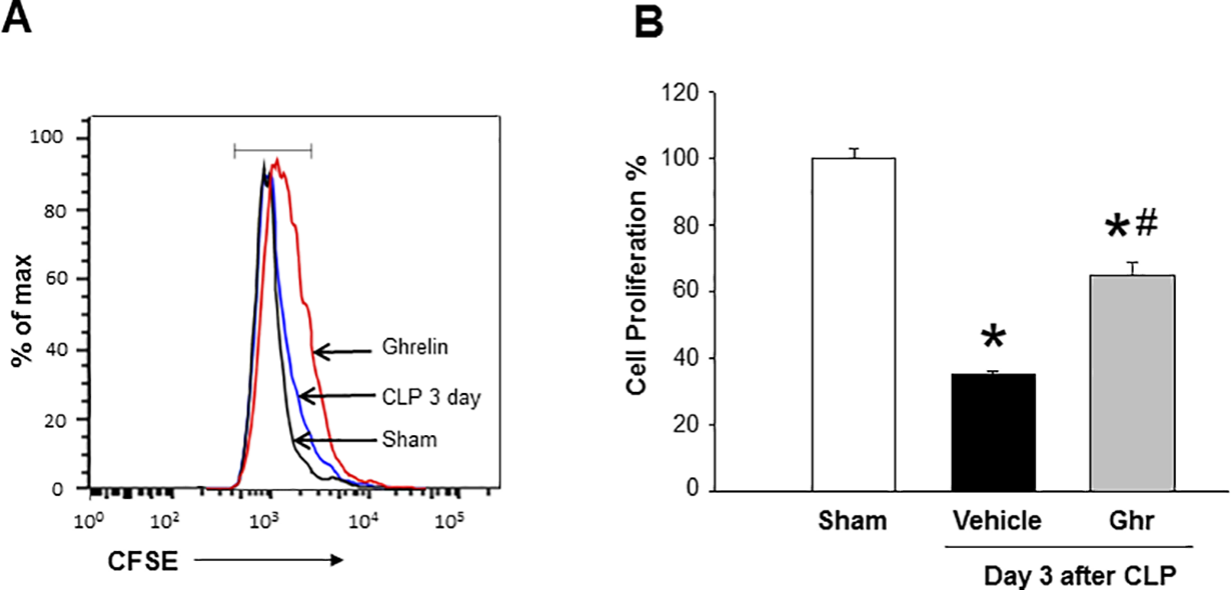
Ghrelin restores CD4 T cell proliferation after sepsis. Mice were subjected to sham operation or CLP with vehicle (normal saline) or ghrelin (2 nm/mouse) injections at 5 and 20 h following CLP operation. Spleens were harvested at day 3 after CLP. Isolated splenocytes were labeled with CFSE (5 μM) and cultured ex vivo in anti-CD3/CD28 (1 μg/ml each) coated wells for 72 h, followed by the staining of cells with APC- anti-mouse CD4 Abs and acquision of the data using flow cytometry. The cell proliferation percentages were calculated and sham group was normalized as 100%. Data are expressed as mean ± SEM (n = 4–5 mice/group). *P < 0.05 vs. sham and ^#^P < 0.05 vs. vehicle-treated animal. CLP, cecal ligation and puncture; CFSE, carboxyfluorescein succinimidyl ester; CD, cluster of differentiation.

At day 3 after CLP, splenocytes were isolated from sham, vehicle- and ghrelin-treated animals for the assessment of CD4 T cell proliferation *ex vivo*. The proliferation of CD4 T cells isolated from the spleen of day 3 CLP-operated mice was reduced by 65% as compared to that of sham mice (Fig 6A and 6B). However, treatment of septic mice with ghrelin significantly increased the proliferation of CD4 T cells by 85% as compared to vehicle-treated mice *ex vivo* (Fig 6B). Thus, ghrelin treatment markedly restored CD4 T cell proliferation in the spleen during sepsis.

### Treatment with ghrelin maintains homeostatic expression of cell cycle proteins in the spleen during sepsis

**Fig 7:**
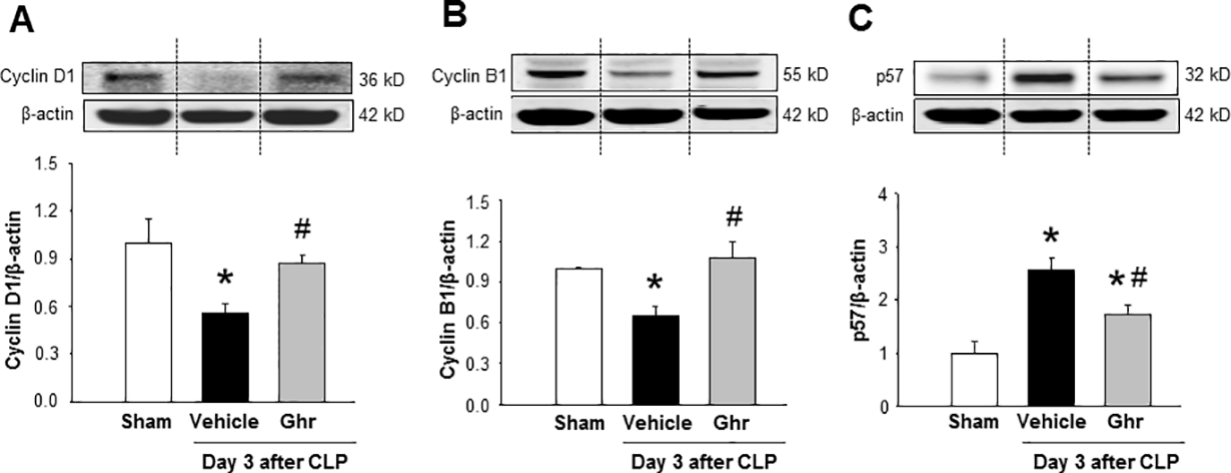
Ghrelin modulates the expression of cell cycle regulatory proteins in sepsis. Mice were subjected to sham or CLP operation and treated with vehicle (normal saline) or ghrelin (2 nm/mouse) at 5 and 20 h following CLP operation. Spleens were harvested at day 3 after CLP. The levels of (A) cyclin D1, (B) cyclin B1, and (C) p57 were measured by western blotting. Data are expressed as mean ± SEM (n = 4–5 mice/group). *P < 0.05 vs. sham and ^#^P < 0.05 vs. vehicle-treated animal. *T*he dotted lines on the representative Western blot reflect the corresponding groups as shown in the bar diagram bellow. CLP, cecal ligation and puncture.

As we noticed the expression of cell cycle regulatory proteins in the spleen was most noticeably altered at day 3 after CLP, we focused to assess the effect of ghrelin on the expression of cell cycle regulatory proteins on day 3 of CLP-operated mice. We found that the expression of cell cycle promoting proteins cyclin D1 and cyclin B1were significantly reduced in vehicle-treated septic animals at day 3 after CLP (Fig 7A and 7B). Conversely, the ghrelin treatment significantly increased the expression of cyclin D1 and cyclin B1 in the spleen by 56% and 67%, respectively, as compared to vehicle-treated septic animals (Fig 7A and 7B). On the other hand, the expression of p57 was upregulated remarkably in the spleen at day 3 after sepsis, while the injections of ghrelin in CLP mice significantly reduced the expression of p57in the spleen by 33% as compared to vehicle-treated mice (Fig 7C). These results clearly demonstrated the role of ghrelin for improving CD4 T cell proliferation by restoring the expression of cell cycle positive regulators and inhibiting the expression of negative regulator in spleen during sepsis.

### Treatment with ghrelin increases AKT activation in the spleen during sepsis

**Fig 8:**
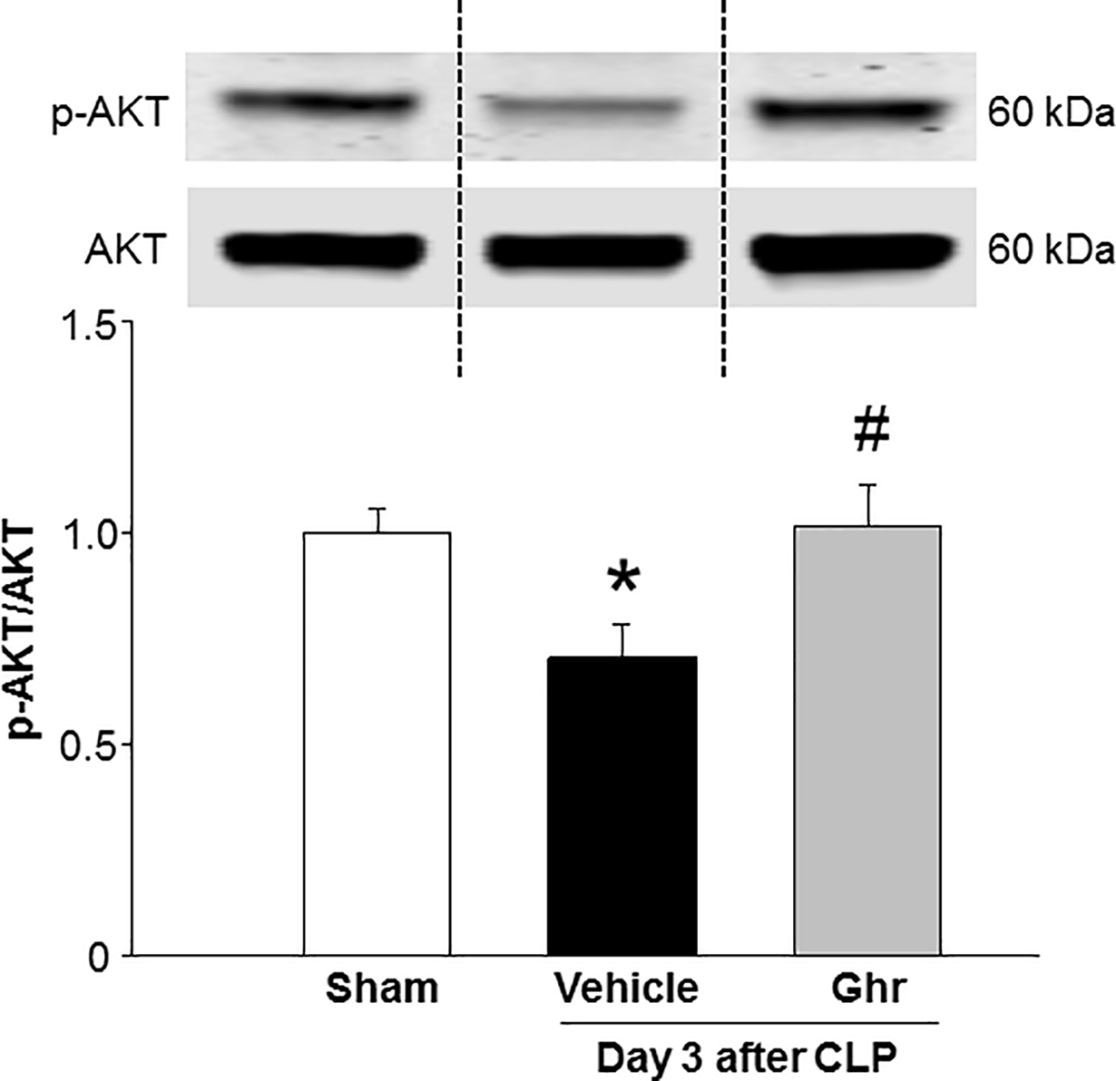
Ghrelin increases AKT phosphorylation in sepsis. Mice were subjected to sham or CLP operation and treated with vehicle (normal saline) or ghrelin (2 nm/mouse) at 5 and 20 h following CLP operation. Spleens were harvested at day 3 after CLP. The levels of phosphorylated AKT were measured by Western blotting. Total AKT served as control. Data are expressed as mean ± SEM (n = 4–5 mice/group). *P < 0.05 vs. sham and ^#^P < 0.05 vs. vehicle-treated animal. The dotted lines on the representative Western blot reflect the corresponding groups as shown in the bar diagram bellow. CLP, cecal ligation and puncture.

AKT signaling pathway plays pivotal role in regulating cell cycle [30]. We therefore assessed the phosphorylation of AKT in the spleen of vehicle- and human ghrelin-treated mice in sepsis. Similar to a recent finding on the p-AKT level in the spleen of murine CLP-induced sepsis [31], we also found that sepsis significantly downregulated p-AKT level in the spleen, while the mice treated with human ghrelin significantly increased p-AKT level by 21% as compared to the vehicle-treated septic mice (Fig 8).

## Discussion

In line with our previous study utilizing a combined treatment strategy with human ghrelin and GH to mitigate sepsis in aged septic animals [22], in the current study we primarily focused on the effect of human ghrelin alone to ameliorate sepsis in young mice. Here, we demonstrated the beneficial role of ghrelin in young septic animals in terms of reversing the deficit of CD4 T cells in spleen during sepsis through the restoration of their maladaptive proliferative ability. We also found that the increased rate of CD4 T cell proliferation in sepsis following treatment with ghrelin was directly linked with the increased expression of cell cycle positive regulators cyclin D1 and cyclin B1 and decreased expression of the cell cycle negative regulator p57. This ultimately led to maintain fine-tuned immune response against infection necessary to reverse the immunosuppression stage that occurs at the late stage of sepsis.

Sepsis is associated with decrease in the frequencies and numbers of CD4 T cells [8, 9, 12]. The decrease of CD4 T cell contents was observed at 12 h following CLP and continued to 3 days or longer after sepsis [8, 11, 15, 32, 33]. Our current results also showed a significant loss of CD4 T cells at day 1 after CLP and sustained this loss at day 3 after sepsis. According to animal-based studies and postmortem tissue samples from septic patients, apoptosis play a pivotal role in the reduction of immune cell numbers during sepsis [8, 9, 12]. The inhibition of lymphocyte apoptosis by various pharmacological or genetic strategies improves the outcomes of sepsis in experimental animal models [34, 35]. In addition to excessive cellular apoptosis, studies have shown that impairment of cell proliferation is also associated with the decreased numbers of immune-reactive cells in sepsis [8, 32, 36]. Our group previously demonstrated the CD4 T cells profiles in septic mice from 5 h to 3 days after CLP and found that the decrease of CD4 T cells in sepsis was accompanied by a gradual reduction of CD4 T cell proliferation rates [8]. Carson *et al*. also demonstrated that sepsis caused a reduction of CD4 T cell contents in mice which was associated with the decrease of proliferative capacity and altered gene expression that even remained at day 14 after CLP [32]. Thus, it appears that both apoptosis and impairment of cell proliferation contribute to the reduction of CD4 T cell numbers during sepsis.

In the current study, we carried-out an *ex vivo* experiment to assess CD4 T cell proliferation; in parallel we also assessed the expression of apoptosis inducing proteins in spleen at multiple time points after CLP in order to better understand about these two events in sepsis. Our data showed that pro-apoptotic proteins, Fas, Fas-L and cleaved caspase-8 were remarkably upregulated at day 1 after CLP, but declined to near sham levels at day 3 after sepsis. On the other hand, CD4 T cell proliferation was slightly decreased at day 1 after sepsis, while it was remarkably reduced on day 3 after CLP as compared to day 1 CLP samples. Our results suggested that the depletion of CD4 T cells in early stage of sepsis was occurred through excessive apoptosis, while in late sepsis (*i*.*e*. 3 day after CLP) impairment of CD4 T cell proliferation could be the cause of decreased contents of CD4 T cells in spleen. In line with this murine model of sepsis, clinical data from freshly isolated lymphocytes of critically ill septic patients showed a high degree of apoptosis at earlier stage of sepsis [37]. Patients’ data also showed that CD4 T cell count increased significantly in sepsis survivors between day 1 and day 6 after sepsis, while this increase was not observed in sepsis non-survivors [37]. This strongly suggests that the level of proliferation of CD4 T cell plays an important role in sepsis outcome. Thus, prevention of the loss of CD4 T cells is an effective therapeutic approach to improve the immune function and reduce the mortality in sepsis. To achieve this, both anti-apoptosis and augmentation of cell proliferation strategies could be helpful for sepsis treatment. Our findings suggest an important notion that anti-apoptotic therapy should be given in the early stage of sepsis while the therapies for augmenting immune cell proliferation is needed at the late phase of sepsis.

The effect of ghrelin on modulation of immunity and inflammation has been demonstrated in previous studies [20, 21, 23, 24]. The decrease of ghrelin and ghrelin receptor with aging is associated with the involution process of thymus [23]. Infusion of ghrelin increases the number of T cells in thymus and peripheral in aged animals [24]. Ghrelin also improves T cell receptor diversity of peripheral T cell subsets in aged animals which could improve immune function [24]. Koo *et al*. showed that injection of ghrelin analogue promoted cell proliferation in spleens and thymus and enhanced immune function [38]. Studies have suggested that the effect of ghrelin on thymic cell proliferation is independent of growth hormone/insulin-like growth factors (IGF-1) axis as ghrelin infusion failed to induce an increase in IGF-1 levels [23]. Ghrelin can dose-dependently increase the proliferation of CD4 T cells *in vitro* [39]. In context of sepsis, the role of ghrelin on CD4 T cell proliferation was not clearly known. The decrease of CD4 T cell proliferation in sepsis was restored by injection of ghrelin into septic animals, suggesting ghrelin plays an important role to maintain CD4 T cell numbers. Our finding on ghrelin-mediated induction of proliferation of CD4 T cells isolated from the septic mice spleen also confirmed the immunostimulatory role of ghrelin in sepsis. The effect of ghrelin on CD4 T cell proliferation is dependent on ghrelin receptor as ghrelin fails to influence CD4 T cell proliferation when using ghrelin receptor deficient mice [39]. Immune cells express ghrelin as well as ghrelin receptor [23, 39]. Since the young mice ubiquitously express ghrelin receptor while the old mice do not [20, 22], the ghrelin-mediated induction of CD4 T cell function could be generated through ghrelin receptor.

Ghrelin has anti-inflammatory role in sepsis and other disease conditions [18, 21, 40, 41]. It is still not completely understood whether the effect of ghrelin on CD4 T cell proliferation is generated directly or indirectly. Koo *et al*. demonstrated the proliferative effect of ghrelin or its analogues on thymus T cells was only observed under *in vivo*, but not *in vitro* condition [38]. The anti-inflammatory effect of ghrelin is mainly mediated through vagus nerve as vagotomy diminishes the protective effect of ghrelin on septic animals [42]. There are anatomic and physiological connections between the central nervous system and the immune system, including 'hardwiring' of sympathetic and parasympathetic nerves to lymphoid organs [43, 44]. Ghrelin can cross blood brain barrier and thus peripheral ghrelin can be transported into the brain [45]. Central administration of ghrelin influences splenic sympathetic nerve discharge, suggesting the effect of ghrelin could be through neural-immune interactions [46]. In addition, sympathetic and parasympathetic nervous system can modulate CD4 T cell activity [47, 48]. Neurotransmitters and neuroendocrine hormones can also modulate immune activity [44, 48]. Thus, the effect of ghrelin on maintaining CD4 T cell proliferation may be through the output of central nerve system. However, Lee *et al*. showed that, under cell culture condition ghrelin signaling on T cell activation and proliferation is mediated through direct activation of ERK1/2 and AKT signaling pathway [39]. It needs to be further investigated whether the effect of ghrelin on CD4 T cell proliferation is solely regulated by central nerve system or by both brain and direct effect of ghrelin on cells.

In the current study, we found ghrelin promoted CD4 T cell proliferation associated with modulation of the cell cycle regulatory proteins. When cells enter the cycle from resting G_0_ phase, cyclin D is a key protein involved in phosphorylation and consequent inactivation of the cell cycle inhibitor retinoblastoma protein [28, 49]. We examined splenic levels of cyclin D1 and D2 after sepsis. Cyclin D1 was markedly decreased after sepsis with the lowest levels at day 3 after CLP in our study. However, there was no change in the expression of cyclin D2 in the same animals after sepsis (data not shown). Ghrelin markedly restored cyclin D1 protein expression in septic animal at day 3 after CLP. Cyclin E is required for the transition from G_1_ to S phase of the cell cycle to initiate DNA duplication [28]. However, we did not observe significant changes on cyclin E1 and E2 levels in septic animals and ghrelin treatment had no effect on their levels (data not shown). Cyclin B is the key regulatory protein controlling mitosis in all eukaryotes [50]. Downregulation of cyclin B prevent a cell enter mitosis phase [28]. Our data revealed that cyclin B1was not altered at day 1 after CLP but significantly reduced at day 3 after sepsis, which is correlated with lower proliferation of CD4 T cells at day 3 after CLP. Treatment of ghrelin completely restored cyclin B1 levels in septic animals at day 3 after CLP. These results indicate that ghrelin can modulate cyclin D1 and B1 levels in septic animals. We also investigated the expression of cell cycle negative regulator, CDK inhibitors p57 in our model. We found that p57 protein expression increased more than 2 folds at day 2 and 3 after CLP. Ghrelin treatment dramatically inhibited p57 expression at day 3 after CLP. Thus, ghrelin restored CD4 T cell proliferation through upregulation of cyclin D1 as well as B1, and downregulation of p57 in septic animals. It has been known that ERK1/2 and AKT signaling pathways can regulate cell cycle [30]. Ghrelin has reported to activate ERK1/2 and AKT signaling pathways, via upstream activation of phosphatidylinositol-3-kinase the kinases PI3K/AKT and MAPK on T cells [28, 39]. We found ghrelin treatment increased AKT activation in spleen of septic animals. Thus, the effect of ghrelin on CD4 T cell proliferation is associated with the activation of AKT pathway.

Here, we assessed the cell cycle regulatory proteins in total splenocytes, thus may not truly represent the change on CD4 T cells in the spleen. However, ghrelin playing major role in thymopoiesis has been reported in previous studies where infusion of ghrelin increases the number of T cells in the thymus and blood [23, 24]. In our previous study, we showed that in mice spleen about 30–35% of total cells are T cells and among them 70% are naïve CD4 T cells [11]. We therefore suggested that the considerable portion of the splenic cyclin D1, B1 and p57 expression could be derived from CD4 T cells. Future studies on the detection of these cell cycle regulatory proteins in purified CD4 T cells will provide more deep insights into the role of ghrelin on murine CD4 T cell proliferation in sepsis.

We previously demonstrated the beneficial outcomes in the survival of septic rats treated with ghrelin over vehicle by monitoring the survival of animals up to 10 days [18]. In line with this finding, Chorny *et al*. also reported beneficial outcomes in the survival of mice with sepsis after treatment with ghrelin by monitoring them for 10 days [51]. Since ghrelin-mediated improvement in the survival outcomes in murine model of sepsis have already been reported, we therefore did not re-perform the survival study for 10 days to re-confirm the ghrelin-mediated survival benefits in mice during sepsis. However, we grossly monitored the survival of the animals between vehicle- and ghrelin-treated groups during 3 days of CLP-induced sepsis and found significant improvement in the survival outcome (100% survival) of the ghrelin-treated septic mice over vehicle-treated mice (50% survival) at day 3 of CLP (data not shown).

In the current study, our approach of using human ghrelin helps us move to the next level of clinical trials for implementing ghrelin as a novel therapeutic agent in septic patients. Ghrelin has highly conserved sequences with huge sequence homology among human, mouse and rats. Mouse and rat ghrelin are 100% identical [52], while the homology between human and mouse or human and rat ghrelin is 93% [52]. Ghrelin from mouse, rat or human sources were all shown to play protective role in rodent sepsis [20–22]. Besides our present finding of the beneficial role of human ghrelin in septic mice, we recently showed human ghrelin also mitigated injury and mortality in cerebral ischemia and whole body radiation injury in rats [53, 54]. As we know that ghrelin binds to its receptor GHSR-1a which is ubiquitously expressed in various cells, there may be a concern whether or not human ghrelin can cross-react with murine ghrelin receptor while administrated into mice. Using protein sequence blast tool of the National Center for Biotechnology Information (NCBI), we confirmed that human and mouse GHSR-1a exhibit 96% homology with each other, indicating human ghrelin works in mice through murine GHSR-1a.

Ghrelin has been administered in humans in multitude of studies and has a favorable safety profile with no side effects [55, 56]. Our current study clearly revealed the immuno-protective function of ghrelin in murine model of sepsis. Thus, ghrelin can be developed as a promising therapeutic agent in sepsis.
